# Combining Machine Learning and Urine Oximetry: Towards an Intraoperative AKI Risk Prediction Algorithm

**DOI:** 10.3390/jcm12175567

**Published:** 2023-08-26

**Authors:** Lars Lofgren, Natalie Silverton, Kai Kuck

**Affiliations:** 1 Department of Biomedical Engineering, University of Utah, Salt Lake City, UT 84112, USA; kai.kuck@hsc.utah.edu; 2 Department of Anesthesiology, University of Utah, Salt Lake City, UT 84112, USA; natalie.silverton@hsc.utah.edu; 3 Geriatric Research, Education and Clinical Centre, Veteran Affairs Medical Center, Salt Lake City, UT 84112, USA

**Keywords:** urinary oxygenation, acute kidney injury, machine learning, cardiac surgery

## Abstract

Acute kidney injury (AKI) affects up to 50% of cardiac surgery patients. The definition of AKI is based on changes in serum creatinine relative to a baseline measurement or a decrease in urine output. These monitoring methods lead to a delayed diagnosis. Monitoring the partial pressure of oxygen in urine (PuO_2_) may provide a method to assess the patient’s AKI risk status dynamically. This study aimed to assess the predictive capability of two machine learning algorithms for AKI in cardiac surgery patients. One algorithm incorporated a feature derived from PuO_2_ monitoring, while the other algorithm solely relied on preoperative risk factors. The hypothesis was that the model incorporating PuO_2_ information would exhibit a higher area under the receiver operator characteristic curve (AUROC). An automated forward variable selection method was used to identify the best preoperative features. The AUROC for individual features derived from the PuO_2_ monitor was used to pick the single best PuO_2_-based feature. The AUROC for the preoperative plus PuO_2_ model vs. the preoperative-only model was 0.78 vs. 0.66 (*p*-value < 0.01). In summary, a model that includes an intraoperative PuO_2_ feature better predicts AKI than one that only includes preoperative patient data.

## 1. Introduction

The early detection and diagnosis of acute kidney injury (AKI) remain an unsolved problem in cardiac surgery. Up to 50% of cardiac surgery patients develop post-operative AKI, which leads to increased mortality, intensive care unit length of stay, and costs [1,2]. The Kidney Disease Improving Global Outcomes (KDIGO) criteria helped standardize the definition of AKI across clinical care and research but, like the previous definitions, rely on changes in serum creatinine and urine output [3]. These markers are meant to reflect kidney function and may not be indicators of early renal cell damage. For example, renal function, measured by glomerular filtration rate (GFR), must drop by 50% before there is a noticeable change in serum creatinine [4]. Also, serum creatinine does not rise until 24–72 h after injury [5,6]. The urine output criteria rely on detecting decreased urine output over a minimum of 6 h. In addition, the optimal urine output and duration thresholds for defining AKI are the subject of debate [7,8]. While a recent study showed that including the urine output criteria in the definition leads to an earlier diagnosis, both the serum creatinine and urine output tracking methods cause a significant delay in AKI diagnosis [9]. This is particularly problematic because as others have indicated, the development of AKI following cardiac surgery is likely related to injury that occurs intraoperatively [10]. This delayed diagnosis precludes injury prevention.

Currently, there are no available clinically validated bedside monitors to assess AKI risk. Clinical tools primarily rely on prediction models to detect patients at risk for AKI before surgery. These risk scores are predominantly based on preoperative risk factors. For example, the Cleveland Clinic Score, which is the most widely studied prediction model, is based on risk factors, such as elevated preoperative creatinine, insulin-dependent diabetes status, and surgery type, among others [11]. Several studies have demonstrated that the Cleveland Clinic Score performs best for predicting post-operative AKI compared to other existing prediction scores [12]. However, one of the limitations of the Cleveland Clinic Score and other widely available risk prediction models is that they were primarily created to detect cases of severe AKI, which require dialysis. In addition, many of the models were based on small cohort sizes and have not been externally validated [13,14]. In addition, these models are static and do not consider intraoperative hemodynamic changes and events. Thus, there is a need for novel methods to dynamically assess the AKI risk status of patients during cardiac surgery. 

As an alternative to risk prediction models, research has emerged to support the use of partial pressure of oxygen in urine (PuO_2_) for the early detection of AKI. Despite the complex etiology of AKI, renal hypoxia is thought to be a common pathway to injury [15]. While there are various pathophysiological processes that lead to injury, such as vasoconstriction or vasodilation, hypovolemia or direct tubular toxicity related to nephrotoxic agents, renal hypoxia is a driver of the disease [16]. It is not currently clinically feasible to continuously measure renal tissue oxygenation. However, others hypothesized that PuO_2_ reflects renal tissue oxygenation. Recent work has demonstrated that an optical fluorescence sensor can be used to reliably measure PuO_2_ and that there is a statistically significant correlation between renal tissue oxygenation and bladder PuO_2_ [16]. In addition, several studies have shown that PuO_2_ in the bladder can be an early indicator of AKI risk in the setting of cardiac surgery [17]. Similarly, we recently developed a noninvasive PuO_2_ monitor that connects to the outlet of the urinary catheter and showed that patients who develop AKI have lower noninvasive PuO_2_ after the termination of cardiopulmonary bypass support [18]. The next step towards the clinical use of PuO_2_ monitoring is to develop a method or algorithm to interpret PuO_2_ in an actionable way. One way to do this is to combine intraoperative PuO_2_ monitoring and preoperative patient information into a single algorithm to monitor the AKI risk status of the patient dynamically. This study aimed to understand how well a machine learning algorithm that includes features derived from real-time noninvasive PuO_2_ monitoring and preoperative patient data could predict post-operative cardiac surgery AKI compared to an algorithm based solely on preoperative risk factors. We hypothesized that an algorithm that included intraoperative PuO_2_ information would have a higher area under the receiver operator characteristic curve (AUROC) than a model trained with preoperatively available patient data alone. 

## 2. Materials and Methods

**Figure 1:**
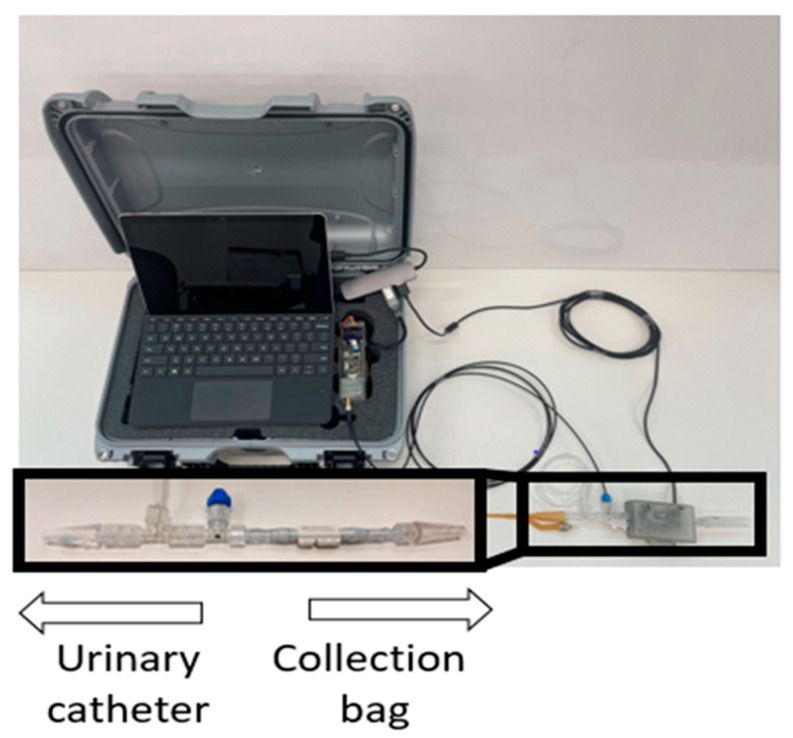
An image of the noninvasive monitor and data collection box that was used to gather PuO_2_ data. The disposable monitor connects between the catheter and collection bag.

We completed a secondary analysis of previously collected clinical data to determine if the algorithm that included information related to intraoperative PuO_2_ monitoring could predict the subsequent development of AKI better than a model that only included preoperative data. The data collection and processing methods have been previously reported [19,20,21]. In summary, after IRB approval, an observational study of 100 cardiac surgery patients who were at high risk for developing AKI based on the Cleveland Risk Score was conducted at the University of Utah Health Sciences (Salt Lake City, UT, USA). Following informed consent, one end of a sterile noninvasive PuO_2_ monitor was connected to the outlet of the urinary catheter, while the other end was connected to the tubing of the urinary collection bag. The device included a thermal-based flow sensor to measure urine flow rate and a temperature probe to correct for the confounding effect of changes in temperature on the raw optical signal used to measure PuO_2_. Each sensor was sampled at 1 Hz, and the device was removed 24 h after the end of cardiopulmonary bypass (CPB). The PuO_2_ monitor and data collection box are shown in Figure 1. Patient demographic information was collected during enrollment and from patient records. 

Several algorithms were used to process the data from the noninvasive monitor to discard data collected during situations related to known unreliable measurements. For example, these algorithms accounted for periods of retrograde urine flow, stagnant urine due to no flow, and measurement error based on the error code output of the thermal-based flow sensor. Additionally, since previous studies have shown that there is a greater amount of oxygen exchange between the urine and the urinary tract tissue at low urine flow rates, PuO_2_ measurements during periods of urine flow <0.5 mL/kg/h were discarded [22]. The intraoperative data were also separated into different time periods related to surgical events. The pre-CPB period was defined as the time between the start of the surgery and the start of the first run of CPB. The CPB period was defined as the entire period between the first run of CPB and the ending of the last run of CPB. The post-CPB period was defined as the period between the end of the last run of CPB and the end of the surgery, marked by when the patient was transferred to the ICU. If more than 70% of the data for a given patient were missing during a time period, the data for that whole time period were not included in the analysis. Patients who received a left ventricular assist device were excluded from this analysis. 

Two different types of potential features were identified for training the machine learning algorithms: preoperatively available patient demographic information and features that could be derived from a real-time PuO_2_ monitor. Potential preoperative available patient demographic features were identified based on previous studies that identified risk factors for developing AKI and included age, sex, body mass index, type of procedure, baseline creatinine, insulin-dependent diabetes status, and whether the patient’s left ventricular ejection fraction was less than 35% [23]. We identified potentially useful features that could be derived from intraoperative PuO_2_ measurements based on a priori knowledge. Based on our prior work showing that there was a difference in mean PuO_2_ during the post-CPB period for patients with and without AKI, mean PuO_2_ for each time period was determined to be a potential feature. We also believed it would be valuable to describe the distribution of the PuO_2_ signal, so the 25th and 75th quartiles, and the median during each time period were determined to be potential features. Others have hypothesized that the relationship between PuO_2_ and subsequent development of AKI is based on cumulative exposure of renal hypoxia, estimated by low PuO_2_ relative to some unidentified threshold [24]. So, the time spent below various PuO_2_ thresholds was calculated. It is also reasonable to suggest that the development of AKI may be related to the cumulative depth of the exposure, so the area below the same thresholds was also calculated. In total, we identified 40 potential features. If a subject was missing data for any of the identified features, data from that subject were not included in the model and analysis.

First, to compare the identified preoperative patient characteristics and known intraoperative risk factors between those that did and did not develop AKI, we used a two-sample chi-square test for categorical variables or an independent sample t-test for continuous variables. Second, we selected features and trained and tested the various models. Due to the limited size of our dataset, we decided to use an automated forward variable selection method to determine the best preoperative features for the two models. We fit a logistic regression model for each individual preoperative feature and determined the *p*-value for each model. We chose the feature with the smallest *p*-value if that *p*-value was <0.05. This feature was retained in the model, and the process was repeated for the remaining features. This process was repeated until all the retained features had a *p*-value < 0.05. All features retained in the automated variable selection model were then used to build the preoperative-only machine learning model. Next, we determined the best PuO_2_-based feature to include with the preoperative features. We chose to include a single PuO_2_-based feature because all PuO_2_-based features were derived from the same signal, and we did not want to introduce potential noise in the feature set. To determine the best individual feature based on the PuO_2_ signal, we calculated the AUROC of each PuO_2_-based feature. The individual predictor with the largest AUROC was combined with the preoperative features to make up the PuO_2_ model. 

**Figure 2:**
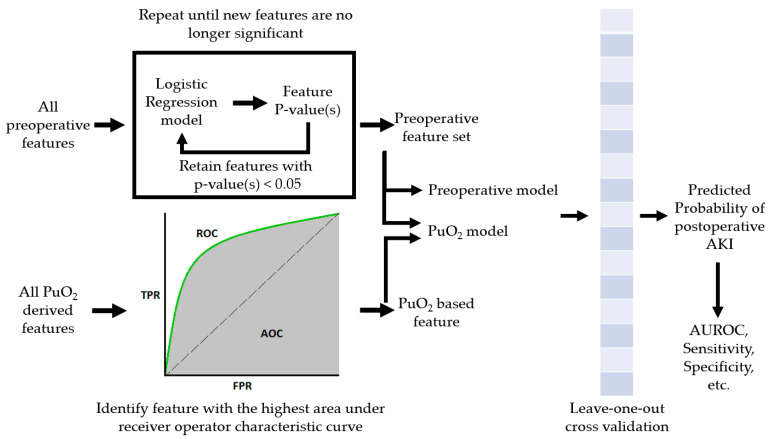
A graphical description of the process for identifying features and training the final machine learning models.

After building our feature dataset, we selected the machine learning model and defined the approach to train and test the different combinations of features. As the dataset we were using to train and test the PuO_2_ and preop model was relatively small and noisy, we chose to use a bootstrap aggregating model, commonly called bagging, because it is an effective method for reducing the risk of overfitting [25]. Bagging fits several base classifiers on random subsets of the data, then aggregates their predictions to make a final prediction. As the support vector machine algorithm has been shown to work well on both linear and nonlinear classification problems, it was chosen as the base classifier for the bagging algorithm. We used the leave-one-out cross-validation method to properly train, evaluate, and compare the preoperative-only and PuO_2_ models [26]. The i-th observation was excluded from the dataset, resulting in a training set of N-1 observations, where N is the total number of subjects in the analysis. The two different models were trained or fitted on the training set and then used to predict the probability of the test subject developing AKI. This process was repeated until each observation in the total dataset was excluded from the training dataset and used as the test sample. This resulted in a predicted probability that each subject developed AKI for each model. This process is shown in Figure 2. The predicted probabilities were used to generate a receiver operator characteristic (ROC) curve for the individual models. The AUROC was calculated for each model, and DeLong’s test was used to compare the two results statistically. The predicted probabilities were then used to make class predictions. If the predicted probability was greater than 0.5, the class prediction was AKI. The class predictions from each model were used to calculate the confusion matrix. Values from the confusion matrix were then used to calculate sensitivity (true positive/(true positive + false negative) and specificity (true negative/(true negative + false positive). The paired sample McNemar test was used to compare the sensitivity and specificity of the different models.

## 3. Results

As previously reported, of the 86 patients that completed the study, 53 were diagnosed with AKI based on KDIGO criteria [19]. Based on the criteria used to discard subjects in this secondary analysis, 73 patients were included in the total dataset, of which 46 were in the AKI group and 27 were in the group that did not develop AKI. Of these 46 patients who did develop AKI, 7 patients had creatinine-based stage I AKI, 4 had creatinine-based stage II AKI, 6 had creatinine-based stage III AKI, and 29 met the criteria for any stage of AKI based on the KDIGO criteria. Table 1 shows the clinical characteristics for patients who did and did not develop AKI. In the univariate analysis, there was a significant difference in baseline creatinine, body mass index, and insulin-dependent diabetes between the two groups. There was not a significant difference in the intraoperative risk factors relative to post-operative AKI. 

For the initial feature selection model, baseline creatinine was the most significant predictor (*p*-value < 0.001). During the second round of the feature selection process, the only significant feature, in addition to baseline creatinine (*p*-value = 0.02), was insulin-dependent diabetes status (*p*-value = 0.04). While baseline creatinine and insulin-dependent diabetes status remained significant when additional features were added, none of the other features were significant in the third round of the feature selection process. This meant baseline creatinine and insulin-dependent diabetes status were the preoperative features used to train the preoperative machine learning model. For the PuO_2_-based features, the time that PuO_2_ was below 35 mmHg during the post-CPB period had an AUROC of 0.61, which was the highest value compared to the other PuO_2_-based features. This meant that the PuO_2_ model included the best preoperative features (baseline creatinine and insulin-dependent diabetes status), and the time PuO_2_ was less than 35 mmHg during the post-CPB phase. 

**Figure 3:**
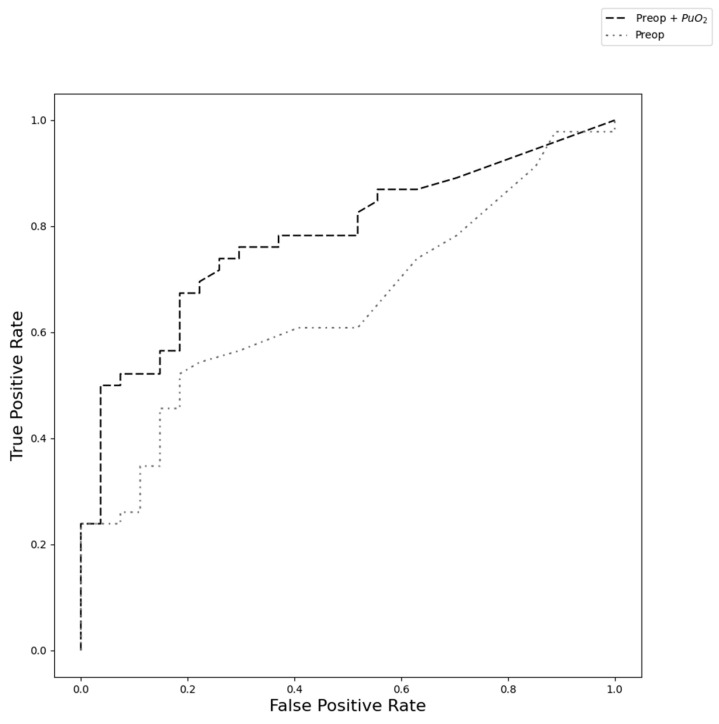
Receiver operator characteristic curves for the PuO_2_ plus preoperative features and preoperative-only models.

The ROC curves are shown in Figure 3. The AUROC for the model that included the preop and PuO_2_-based features was 0.78 (95% CI: 0.62–0.83), whereas the AUROC for the preoperative-only model was 0.66 (95% CI: 0.50–0.70). The AUROC for the combined model was significantly higher than the preoperative-only AUROC (*p*-value < 0.01). There was no statistically significant difference in the specificity of the two models (*p*-value = 0.48, preoperative = 0.85, PuO_2_ = 0.78). In contrast, the PuO_2_ model was statistically more sensitive than the preoperative model (*p*-value < 0.001, preoperative = 0.40, PuO_2_ = 0.72). 

## 4. Discussion

In this study, we found that a machine learning model that includes intraoperative PuO_2_ data and preoperative patient information was a better predictor of AKI than a model that was trained on just preoperatively available patient data alone, based on AUROC values of 0.77 and 0.66, respectively. This difference was the result of the increased sensitivity of the PuO_2_ model compared to the model based on preoperative clinical data alone.

Like the preoperative model in this study, many of the clinically available preoperative risk scores, like the Cleveland Clinic Score, incorporate baseline creatinine and insulin-dependent diabetes status in the final model [14]. The difference is that the other risk prediction models typically include additional features. However, many of these other risk prediction models were derived from feature sets with >30,000 subjects. Additionally, the most common scores were designed only to predict AKI requiring dialysis. Dialysis is a rare event, affecting up to 2% of AKI patients, and usually occurs several days after the operation, which limits the effectiveness of these models [13]. In contrast, this study included only 73 subjects and predicted the development of any stage or type of AKI based on the KDIGO definition. Despite these differences, the AUROC for the preoperative model in this study was similar to the AUROC of 0.66 (95% CI: 0.65–0.68) reported for the Cleveland Clinic Score in a more recent study that looked at a separate validation cohort [27]. 

New risk prediction scores have been developed to predict AKI outside of cases requiring dialysis based on the KDIGO definition. For example, Birnie et al. found that their model had an AUROC of 0.74 and better predictive performance than several common risk scores, including the Cleveland risk score, when using the KDIGO AKI definition [13]. While the score is available as an online calculator, similar to other risk prediction models, it still does not take into account intraoperative variables. This means this and other risk prediction scores are not useful for dynamic monitoring of the patient. Since these scores do not provide dynamic monitoring of the status of the patient, the care team cannot use them as tools to guide intervention or resuscitation. In addition, these models identify patients at high risk but are based almost exclusively on non-modifiable risk factors, limiting their usefulness in patient management. In contrast, recent studies have shown that the administration of a diuretic such as furosemide or increasing mean arterial pressure by administering vasopressin increases PuO_2_, suggesting that intraoperative urine oxygen may be a modifiable risk factor [28,29,30,31]. This means that, in the future, the clinician or care team may be able to make decisions based on a PuO_2_ monitor that incorporates a risk prediction algorithm. More robust and large-scale studies are needed, however, to validate that these interventions improve PuO_2_ and that increasing PuO_2_ reduces the incidence of AKI. 

This study is not the first study to demonstrate the value of including intraoperative data in risk prediction models for post-operative AKI. Parolari et al. demonstrated that including intraoperative and post-operative data significantly improves the AUROC compared to a model that only uses preoperative data [32]. Tseng et al. demonstrated that a machine learning model trained on 94 features that include preoperative and post-operative measurements to predict post-operative cardiac surgery AKI can achieve an AUROC of between 0.78 and 0.85 depending on the model type [33]. In fact, of the 20 most important features identified in their study, more than half are intraoperative variables. However, many of the intraoperative variables used to train the model are not available until after the surgery is complete, such as the total intraoperative urine output or total intraoperative volume of IV fluids infused. It is also worth noting that many of these variables are stored on paper notes or in the electronic health record. Due to the lack of interoperability between different electronic health records and medical devices, these algorithms may not be useful during surgery. In addition, the intraoperative time-series parameters that were used to train the Tseng et al. model were based on blood pressure and heart rate, which are systemic parameters and are not specific to the kidney. Furthermore, a recent meta-analysis showed that targeting a higher mean arterial pressure during cardiac surgery did not have an effect on the rate of AKI. While the exact reason is unclear, it may be because there are other renal hemodynamic factors in addition to mean arterial pressure, such as blood oxygen content and local tissue micro perfusion, that influence medulla tissue oxygenation. 

Others have explored the use of urinary and serum biomarkers to dynamically assess a patient’s risk for AKI. Studies have found that certain biomarkers of kidney injury, like interleukin 18 (IL-18), kidney injury molecule 1 (KIM-1), neutrophil gelatinase-associated lipocalin (NGAL), liver-type fatty acid-binding protein (L-FABP) as a singular biomarker, and L-FABP in combination with tissue inhibitor of metalloproteinases-2 (TIMP2), increase sooner than serum creatinine following renal injury [21,34,35,36,37,38]. These tests are still limited by long processing times. The only FDA-approved point-of-care test, NephroCheck, requires a minimum of 20 min to obtain a result. Other biomarker tests that have been extensively studied have to be sent to the central laboratory for processing, which often requires expensive equipment and can take hours or sometimes days to obtain a result. Ultimately, the clinical adoption of these biomarker tests has been slow, despite evidence-based recommendations, partially because the cost–benefit ratio of these tests is unclear [39,40,41,42]. 

Others have also demonstrated the predictive value of PuO_2_ intraoperatively. However, it is also worth noting that there may be multiple methods for interpreting PuO_2_ data that may be effective. For example, Zhu et al. found the more time bladder PuO_2_ is below 15 mmHg during cardiac surgery, the more likely a person is to develop AKI [43]. In a previous study, we found that a mean PuO_2_ of less than 25 mmHg during the post-CPB period was associated with the KDIGO definition of AKI and other various definitions of renal dysfunction [19]. Noe et al. showed that a lower nadir value following CPB was associated with a greater risk for developing AKI [18]. To date, we are not aware of any large-scale studies validating the best PuO_2_ threshold and interpretation method for predicting AKI. 

An advantage of using a machine learning approach is that the algorithms are typically good at learning complex, nonlinear relationships. This means the exact threshold for feature choice may not be as important. In the future, a PuO_2_ machine learning model may not require the selection of a threshold and may be able to interpret the PuO_2_ waveform directly. Another important advantage of using machine learning is that the output can be a continuous variable representing an individual’s risk level for developing AKI. The output may not be a binary yes or no prediction but, instead, may indicate the confidence level of the model’s prediction or the overall risk level of the subject in their current state. 

One of the limitations of this study is the small size of the dataset used to train and test the two different machine learning models. Other studies looking at machine learning algorithms and prediction of AKI rely on thousands or tens of thousands of subjects. This is because AKI is likely multifactorial. This means there are many additional features that could increase a person’s risk of developing AKI in addition to the features identified in this study. However, to incorporate all potential features, a large number of subjects would be necessary to properly describe the high-dimensional feature space. This means that due to the small dataset, the size of the models used in this study was limited, both in terms of complexity and the number of features. Thus, we had to adapt a feature selection process instead of including all the identified features. In addition to using a feature selection process, we used leave-one-out cross-validation. This means that the results may not be generalizable for other populations or cohorts. In addition, this was a proof-of-concept study, and more work is necessary to design, test, and validate a robust decision-support tool. 

It is also important to note that, in this study, AKI was defined according to the KDIGO criteria. While previous definitions of AKI included criteria based on an estimated GFR, these were removed in more recent guidelines due to the risk of misclassification due to estimated GFR [44]. While some may still advocate for using estimated GFR as a tool for detecting AKI, we used the KDIGO definition as it is supported by the latest clinical guidelines and is standard across the most recent research studies. 

In summary, we have shown that a model that includes an intraoperative PuO_2_ feature is a better predictor than a model that only includes preoperatively available patient data. These findings imply that intraoperative PuO_2_ monitoring may provide a method for intraoperatively assessing a patient’s risk for developing AKI after cardiac surgery and be used as a tool to guide potential interventions.

## Figures and Tables

**Table 1:** Summary of the clinical characteristics for the patients who did and did not develop AKI. For continuous variables, a t-test was used to compare the populations. For categorical variables, a chi-square test was used.

Feature	AKI (n = 46)	No AKI (n = 27)	*p*-Value
Age (years), mean ± SD	65 ± 11	62 ± 15	0.27
Body Mass Index, mean ± SD	29.38 ± 5.89	26.57 ± 5.32	0.05
Baseline creatinine, mean ± SD	1.12 ± 0.26	0.97 ± 0.25	0.014
Left Ventricular Ejection Fraction <35%, n (%)	7 (15)	2 (8)	0.54
Insulin dependent diabetes, n (%)	14 (30)	1 (3)	0.015
Female, n (%)	14 (30)	8 (30)	0.99
Type of procedure—Isolated CABG	16 (35)	7 (26)	0.60
Type of procedure—Single Valve	9 (20)	6 (22)	0.99
Type of procedure—Single Valve + CABG	7 (15)	3 (11)	0.88
Type of procedure—>1 valve	5 (11)	3 (11)	0.99
Type of procedure—Other	7(15)	7(26)	0.66
Intraoperative risk factors			
CPB Time (min), mean ± SD	160.42 ± 55.28	167.74 ± 70.25	0.62
Transfusion rate, n (%)	26(56)	15(56)	0.99
Urine output (mLs), mean ± SD			

## Data Availability

The data presented in this study are available on request from the corresponding author. The data are not publicly available due to issues related to confidentiality.
